# Research on the slip deformation characteristics and improvement measures of concrete-faced rockfill dams on dam foundations with large dip angles

**DOI:** 10.1038/s41598-024-59222-0

**Published:** 2024-04-15

**Authors:** Jun Gao, Xiaoke Han, Wenchao Han, Faning Dang, Jie Ren, Haibin Xue, Jianyin Fang

**Affiliations:** 1 State Key Laboratory of Eco-hydraulics in Northwest Arid Region, Xi’an University of Technology, Xi’an, 710048 Shaanxi China; 2 School of Civil Engineering and Architecture, Xi’an University of Technology, Xi’an, 710048 Shaanxi China

**Keywords:** Pumped storage power station, Upper reservoir dam, Concrete face rockfill dam, Dam foundation with large dip angle, Slip deformation, Anti-sliding stability, Improvement measure, Energy science and technology, Engineering

## Abstract

The pumped storage power station (PSPS) is an important measure to achieve the strategic goal of “dual carbon”. As one of the preferred types for the upper reservoir dams of PSPSs, the concrete-faced rockfill dam (CFRD) often has a dam foundation on a steep transverse slop and is prone to produce slip deformation along the slope, resulting in poor anti-sliding stability of the dam slope. It is dangerous for the operation safety of PSPSs. Therefore, the slip deformation of CFRDs on dam foundations with large dip angles is investigated. The mechanism for the initiation of slip deformation is revealed. The design measures of physical mechanic and geometric structure are proposed to reduce slip deformation. The results show that the larger sliding forces and smaller anti-sliding forces are the fundamental reasons that CFRDs on dam foundations with large dip angles are prone to produce slip deformation. The larger the dip angle of the dam foundation, the larger the slip deformation of the dam body and face slab, and the smaller the safety factor of the dam slope. When the dip angle of the dam foundation is greater than 15°, the safety factor of the dam slope is less than the minimum value of 1.5 required by codes. The addition of pressure slopes can effectively reduce the slip deformation of the dam body or face slab and significantly improve the anti-sliding stability of the dam slope. When the height or width of the pressure slope platform is greater and the cohesion or internal friction angle of the pressure slope is larger, the slip deformations of the dam body and face slab are smaller, and the safety factor of the dam slope is greater. It is recommended that the height and width of the pressure slope platform be 1/2 times the maximum height of the main dam, and the density (cohesion and internal friction angle) of the pressure slope be equivalent to that of the main dam’s rockfill material. The research results can provide theoretical and technical support for the design and construction of CFRDs for the upper reservoir of PSPSs.

## Introduction

China has become the world’s largest country in terms of energy production. The vigorous development of clean energy is not only an important way to achieve sustainable development and promote energy structure transformation, but also a key measure to achieve the strategic goal of “dual carbon”^1^. With the rapid development of wind power, nuclear power, and solar energy, it is urgent to operate pumped storage power stations (PSPSs), which have multiple functions, such as peak shaving, valley filling, frequency regulation, phase modulation, and emergency standby operation^2^. However, the installed capacity of PSPSs in China accounted for only 1.4% of total installed capacity at the beginning of 2020, and this cannot effectively meet the needs of safe, stable, and economic operation of power systems. The outline of the 14th five-year plan (2021–2025) for national economic and social development and Vision 2035 of the People’s Republic of China clearly proposes to accelerate the construction of PSPSs. As an important part of PSPSs^3^, the upper reservoir dam is usually located on the mountaintop or mountainside of alpine and canyon regions. The terrain and geological conditions of dam sites are extremely complex and particularly suitable for building rockfill dams with local materials, low anti-seepage materials, and good flexibility. The concrete-faced rockfill dams (CFRDs) have gradually become one of the preferred types for upper reservoir dams of PSPSs due to their excellent resistance to seepage, good ability to withstand the effects of earthquakes, and strong adaptability to deformation^4–6^, for example, the upper reservoir dams of PSPSs in Hami, Zhouning, and Fukang.

The deformation characteristics of CFRDs^7–10^ are significantly impacted by the terrain and geological conditions of dam sites. The long-term deformation characteristics of CFRDs in narrow valleys were studied by Deng gang et al.^11^. The results showed that there was an obvious arch effect on settlement deformation. Then, the influence of narrow valley terrain (different bank slopes and riverbed widths) on the deformation characteristics of CFRDs was further studied. The engineering measures that improved the deformation characteristics of CFRDs in canyon regions were explored^12^. The deformation characteristics of CFRDs built on asymmetric valleys were first studied by Dang Faning et al.^13^. Asymmetric valleys tended to cause nonuniform settlement deformation. Improvement measures for adjusting rolling parameters were proposed. The new valley shape parameters^14^ were further defined. The effects of the valley width coefficient, valley slope coefficient, and valley asymmetric coefficient on the deformation characteristics of the dam body were studied. The valley shape had an obvious constraint effect on the dam body, and it increased with the decrease of aspect ratio^15^. The steeper the valley slope was, the more obvious the arch effect^16,17^. The more severe the asymmetry of the valley, the larger the range and value of nonuniform settlement deformation. The excessive deformation of CFRDs may cause problems with the anti-sliding stability of the dam slope. The safety control index of the anti-sliding stability of the dam slope was discussed by Chen Zuyu et al.^18^. The stability of dam slopes was studied by Song Laifu et al.^19^ and Jiang Shuihua et al.^20^ with reliability methods. In addition, the anti-sliding stability of the dam slope was studied by Zhou Jianfeng et al.^21^, Wei Kuangmin et al.^22^, and Lv Qingfeng et al.^23^ via linear and nonlinear strength parameter methods. Wu Zhenyu et al.^24,25^ discussed some problems on the reliability analysis of dam slope stability with nonlinear strength index, and pointed out that the influence of strength index distribution on the reliability analysis of dam slope stability cannot be ignored.

In summary, there are plentiful research results on the deformation characteristics and dam slope stability of CFRDs for complex valley terrain conditions. However, different from CFRDs of normal hydropower stations, the dam foundations of CFRDs for upper reservoirs of PSPSs often have large dip angles, which can easily cause slip deformation and poor anti-sliding stability, seriously endanger the operational safety of CFRDs in the upper reservoir of PSPSs^26^. Meanwhile, there are still few reports on relevant research results. Moreover, the “Design code for concrete face rockfill dams” notes that the stability of the slopes of CFRDs must be studied when adverse terrains occur. In view of this, the slip deformation of CFRDs on dam foundations with large dip angles is investigated. The mechanism for the initiation of slip deformation of CFRDs on dam foundations with large dip angles is revealed. The design measures of physical mechanic and geometric structure are proposed to reduce the slip deformation of CFRDs on dam foundations with large dip angles. The values of the height or width of the pressure slope platform and the cohesion or internal friction angle of the pressure slope are recommended. The research results can provide theoretical and technical support for the design and construction of CFRDs for the upper reservoir of PSPSs.

## Numerical models established for CFRDs on dam foundations with large dip angles

### Numerical models

**Figure 1:**
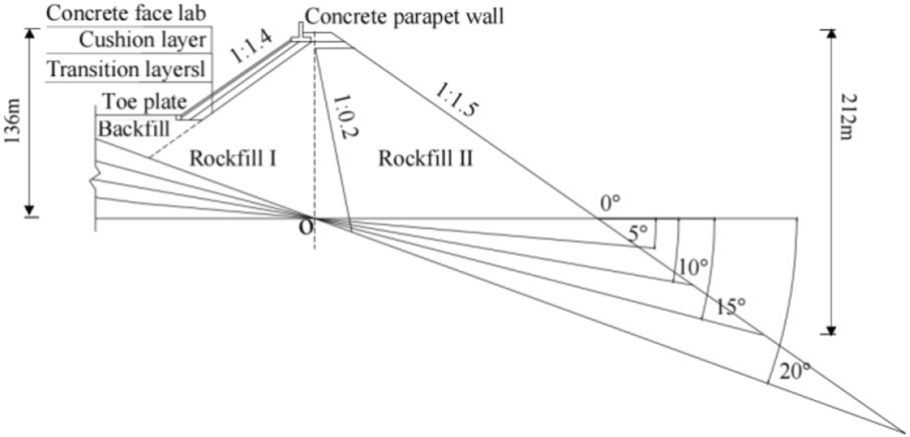
Research plan of the transverse dip angle of the dam foundation.

**Figure 2:**
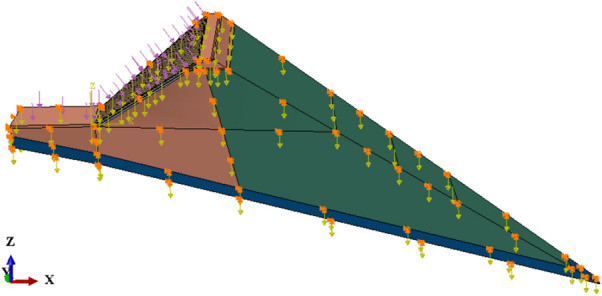
Generalized numerical model of CFRD when the dip angle of the dam foundation is 15°.

**Figure 3:**
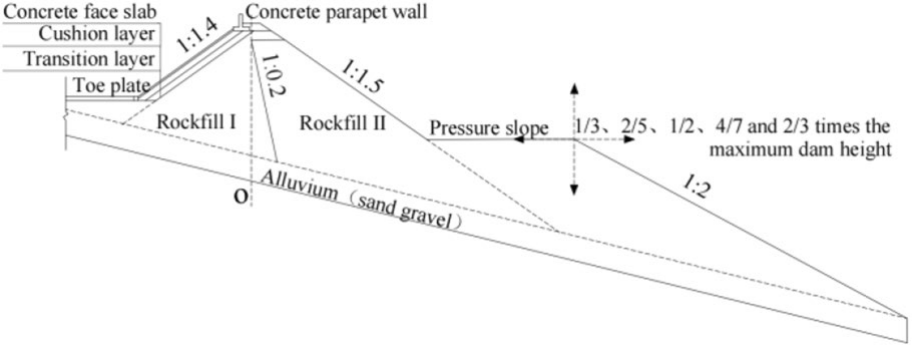
Research plan of pressure slopes on dam foundations with large dip angles.

**Figure 4:**
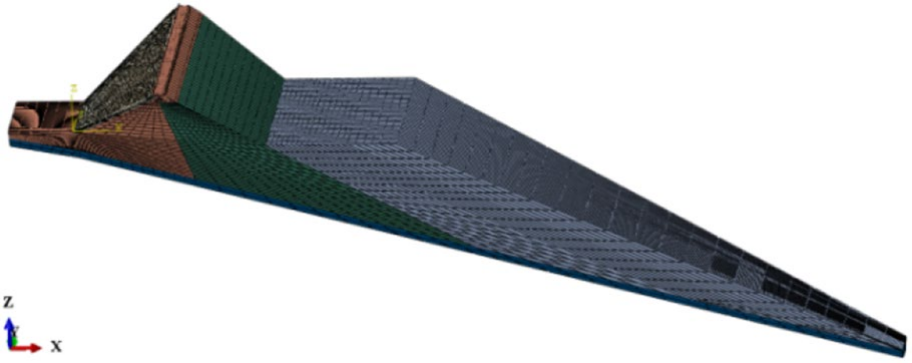
Generalized numerical model of CFRD with pressure slope when the dip angle of the dam foundation is 15°.

The proposed CFRDs are located on U-shaped valleys. The dam heights are both 136 m. When the dip angle of the dam foundation is 15°, the maximum dam height is 212 m. The maximum dam height is defined as the height from the dam bottom to the dam crest. The length of dam axis is 300 m. The width of the dam crest is 10 m. The slope ratios of upstream and downstream dam slopes are 1:1.4 and 1:1.5, respectively. The slope ratios of right and left valleys are 1:0.75 and 1:1.05, respectively. The slope ratio of the boundary between rockfill I and rockfill II is 1:0.2. The top and bottom thicknesses of the concrete face slab are 0.75 m and 1.0 m, respectively. The horizontal widths of the cushion layer and transition layer are both 3.0 m. The dip angles of the dam foundation are taken as 0°, 5°, 10°, 15°, and 20°. The research plan without pressure slopes is shown in Fig. 1. The generalized numerical model without pressure slope is shown in Fig. 2, which is established with reference to the upper reservoir of Fukang PSPS in China (the main dam foundation of the upper reservoir towards downstream is more than 20° and locally exceeds 30°.). ABAQUS is employed for establishing numerical models and calculating three-dimensional deformations in this paper, which is a large-scale commercial finite element software that is widely used in hydraulic engineering and geotechnical engineering. The construction process and boundary conditions are strictly followed. The boundary conditions are that the four sides of models are normally constrained and the bottom of models is fully constrained, as shown in Fig. 2. The type of finite element mesh for CFRD is C3D8R, and the number of finite element mesh for CFRD is 38,200 when the dip angle of the dam foundation is 15°. The research plan with pressure slopes is shown in Fig. 3. The generalized numerical model with pressure slopes is shown in Fig. 4, which is established with reference to the upper reservoir of Fukang PSPS in China. The type and number of finite element mesh for the main dam are consistent with those of non-pressure slopes. The type of finite element mesh for pressure slopes is C3D8R, and the number of finite element mesh for pressure slopes is 10,332. When the dip angle of the dam foundation is 15°, the height and width of the pressure slope platform are 2/3 and 1/2 times the maximum height of the main dam. The friction contact units are set between the concrete face slab and the concrete face slab, between the concrete face slab and the toe slab, between the concrete face slab and the concrete parapet wall, and between the concrete face slab and the cushion layer. The shear stress is calculated by Coulomb’s theory. The formula for the ultimate shear stress is as follows:

1 $$ \tau = \mu P $$

where *μ* is the friction coefficient and *P* is the normal contact pressure. When the tangential friction force exceeds the ultimate shear stress, the slip deformation occurs on the contact surface. The shear, dislocation, and extension deformation of structural joints can be effectively simulated by the Coulomb contact model^27^.

### Material parameters

The mechanical properties of the construction materials of CFRDs are very complex, so it is very important to describe the mechanical properties of construction materials accurately. At present, nonlinear elastic models and elastoplastic models are commonly used to describe the mechanical properties of dam materials. The first type is represented by the E–μ model and E–B model proposed by Duncan–Chang, and the second type is represented by the K–G model proposed by Tsinghua University and the Nanjing Hydraulic Research Institute (NHRI) model proposed by Academician Shen Zhujiang. However, many experiments show that both types of models have limitations. Thus, the improved hyperbolic model and creep model have been proposed. The research results indicate that the horizontal displacement of the dam body calculated by the K–G model is closer to the actual measured value. The vertical displacement of the dam body calculated by the E–μ model and E–B model is closer to the actual measured value. The stress of the dam body calculated by the NHRI model is closer to the actual measured value^28,29^. The nonlinear hyperbolic elastic model (E–μ model or E–B model) is recommended by “Design code of asphalt concrete facings and cores for embankment dams”. The concept of material parameters of E–μ model or E–B model is clear, and the geometric and physical meanings are clear. Moreover, the E–μ model or E–B model is more convenient to be used. Thus, the deformation characteristics of CFRDs are studied with the Duncan‒Chang E–B model^27^. The corresponding parameters of dam materials are shown in Table 1. The strength reduction method^30,31^ (SRM) is selected to study the stability of the dam slope. The SRM continuously reduces the strength of rockfill material until it is damaged. The ratio of the shear strength of rockfill materials to actual shear stress is defined as safety factor (*S*_*F*_, safety coefficient). The basic principle of SRM can be expressed as follows:

2 $$ \tau^{\prime} = \frac{\tau }{{S_{F} }} = \frac{{C + \sigma {\text{tan}}\phi }}{{S_{F} }} = \frac{C}{{S_{F} }} + \frac{{\sigma {\text{tan}}\phi }}{{S_{F} }} = C^{\prime} + \tan \phi^{\prime} $$

where $C^{\prime}$ is the virtual cohesion after reduction, $\phi^{\prime}$ is the virtual internal friction angle after reduction, $\tau^{\prime}$ is the shear strength after reduction; *C* is the cohesion before reduction; *φ* is the internal friction angle before reduction; *τ* is the shear strength before reduction; *σ* is the normal stress on the fracture surface of rockfill material. *S*_*F*_ increases gradually from a small value, and the value of *S*_*F*_ is the safety factor of rockfill material when the rockfill material is in critical failure state.

**Table 1:** Duncan Chang E–B model parameters of dam materials.

Materials	*K*	*N*	*R*_f_	*C*/(kPa)	*φ*/°	Δ*φ*/°	*K*_b_	*m*	*K*_ur_	*ρ* (g/cm^3^)
Rockfill I	1400	0.27	0.67	28	50	11.8	764	0.03	2701	2.07
Rockfill II Pressure slope Backfill	1180	0.28	0.67	24	48	12.2	764	0.03	2276	2.10
Cushion layer	1150	0.28	0.60	21	51	12	623	0.47	2213	2.20
Transition layer	1040	0.28	0.60	21	52	12	623	0.12	2213	2.30
Overburden layer (Alluvium)	700	0.30	0.79	10	46.5	5.8	210	0.28	1400	2.09
Concrete face slab Concrete parapet wall Toe slab	*ρ* = 2.40 g/cm^3^; *E* = 25 GPa; *μ* = 0.20									

where *K* is the modulus number, *n* is the modulus index, *R*_*f*_ is the failure ratio, *C* is the cohesion, *φ* is the internal friction angle, △*φ* is the increment of internal friction angle *φ*, *K*_*b*_ is the bulk modulus number, and *m* is the bulk modulus index, *K*_*ur*_ is the modulus number for unloading and reloading, *ρ* is the density.

Although the location and shape of the most dangerous slip plane of the dam slope cannot be directly identified by SRM, they can be obtained by surface fitting according to the location of the maximum equivalent plastic strain of the critical state^32^. The corresponding parameters of dam materials are shown in Table 2.

**Table 2:** Mohr–coulomb model parameters of dam materials.

Materials	*E* (MPa)	*υ*	*ρ* (g/cm^3^)	*C* (kPa)	*φ*/°
Rockfill I	100	0.35	2.09	30	50
Rockfill II Pressure slope Backfill	90	0.35	2.12	25	48
Cushion layer	110	0.34	2.23	21.5	51
Transition layer	100	0.32	2.32	21.5	52
Overburden layer (Alluvium)	120	0.37	2.09	10	46.5

where *E* is the Young’s modulus, and *μ* is the Poisson’s ratio.

## Slip deformation characteristics of CFRDs on dam foundations with large dip angles

Aiming at the problem of the slip deformation of CFRDs on dam foundations with large dip angles, the influence of the dip angle of the dam foundation on the slip deformation (represented by settlement deformation and deflection deformation) of the dam body or face slab is investigated. The anti-sliding stability of the dam slope for CFRDs on dam foundations with large dip angles is investigated to provide theoretical basis for the study of the mechanism for the initiation of slip deformation of CFRDs.

### Effect of the dip angle of the dam foundation on the slip deformation of the dam body

**Figure 5:**
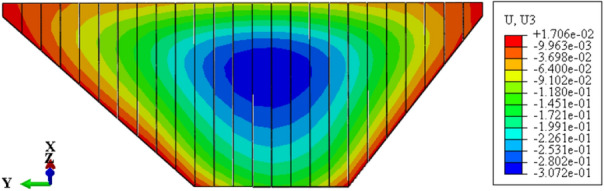
Settlement deformation contour of the concrete face slab when the dip angle of the dam foundation is 15° (unit: m).

**Figure 6:**
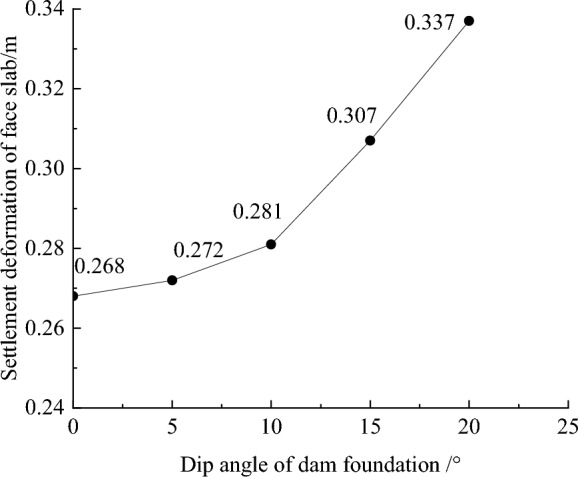
Relationship between the settlement deformation of the concrete face slab and the dip angle of the dam foundation (unit: m).

The settlement deformation contour of concrete face slab for CFRD on the dam foundation with a large dip angle during the normal operation period is shown in Fig. 5, when the dip angle of dam foundation is 15°. The maximum settlement deformation of concrete face slabs is approximately elliptical (controlled by the shape of the river valley), and there is slight discontinuous settlement deformation between concrete face slabs due to the setting of blocks. When the dam foundation is flat (the dip angle of dam foundation is 0°), the maximum settlement deformation of concrete face slabs is approximately 0.5 times the height of concrete face slabs at the vertical direction and approximately 0.5 times the length of the dam axis at the horizontal direction. As the dip angle of the dam foundation increases, the law of deformation settlement for concrete face slabs is basically same, but the gradient of settlement deformation increases, and the discontinuous settlement deformation between concrete face slabs gradually weakens. Figure 6 shows the relationship between the settlement deformation of concrete face slabs and the dip angle of the dam foundation (normal operation period). When the dip angle of the dam foundation is 0°, 5°, 10°, 15°, and 20°, the maximum settlement deformation of concrete face slabs under the co-action of gravity and water pressure is 0.268, 0.272, 0.281, 0.307, and 0.337 m, respectively. The larger the dip angle of the dam foundation, the larger the settlement deformation of concrete face slabs. The settlement deformation of concrete face slabs is mainly controlled by the settlement deformation of the dam body, and the settlement deformation of concrete face slabs when the dip angle of the dam foundation is 20° is approximately 1.3 times that of 0°.

**Figure 7:**
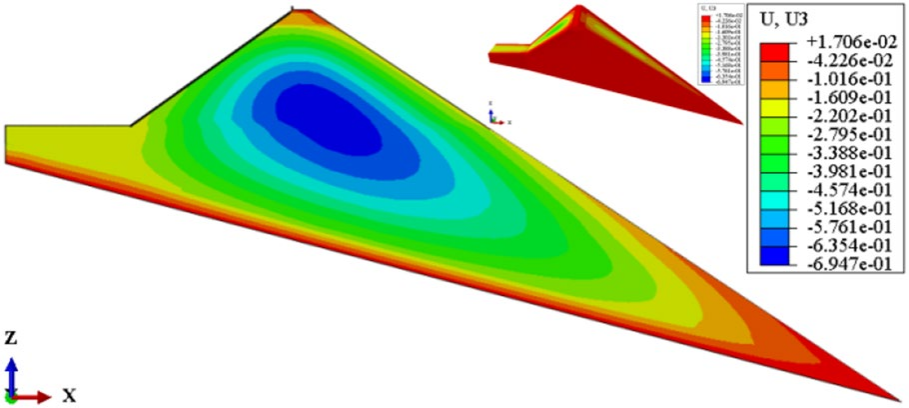
Settlement deformation contour of the dam body when the dip angle of the dam foundation is 15° (unit: m).

**Figure 8:**
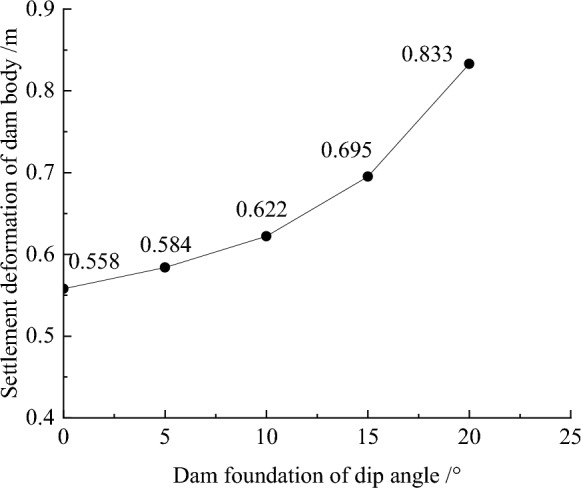
Relationship between the settlement deformation of the dam body and the dip angle of the dam foundation (unit: m).

Figure 7 shows the settlement deformation contour of the dam body for CFRD on the dam foundation with a large dip angle during the normal operation period when the dip angle of the dam foundation is 15°. The settlement deformation of the dam body has an approximately elliptical distribution. When the dam foundation is flat (the dip angle of the dam foundation is 0°.), the maximum settlement deformation is approximately 0.5 times the dam height at the vertical direction and near the dam axis at the horizontal direction. When the dam foundation tilts, the location of maximum settlement deformation decreases vertically and leans laterally downstream. As the dip angle of the dam foundation increases, the location of maximum settlement deformation becomes lower and gradually moves away from the dam axis, and the gradient of settlement deformation increases. Figure 8 shows the relationship between the settlement deformation of the dam body and the dip angle of the dam foundation (normal operation period). When the dip angle of the dam foundation is 0°, 5°, 10°, 15°, and 20°, the maximum settlement deformation of the dam body under the co-action of gravity and water pressure (buoyancy) is 0.558, 0.584, 0.622, 0.695, and 0.833 m, respectively. The settlement deformation does not exceed 1.0% of the dam height. The larger the dip angle of the dam foundation, the greater the settlement deformation of the dam body; the settlement deformation when the dip angle of the dam foundation is 20° is approximately 1.5 times that of 0°.

**Figure 9:**
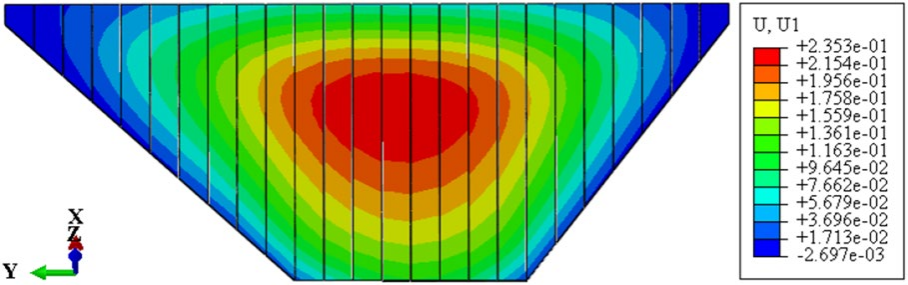
Deflection deformation contour of concrete face slabs when the dip angle of the dam foundation is 15° (unit: m).

**Figure 10:**
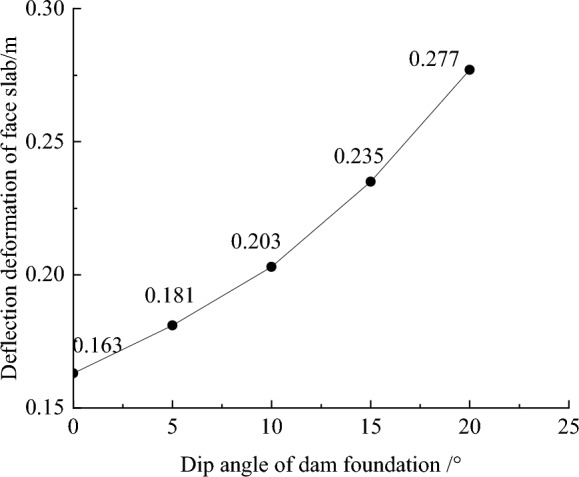
Relationship between the deflection deformation of concrete face slabs and the dip angle of the dam foundation (unit: m).

Figure 9 shows the deflection deformation contour of concrete face slabs for CRFD on the dam foundation with a large dip angle during the normal operation period when the dip angle of the dam foundation is 15°. The deflection deformation of concrete face slabs is approximately elliptical, and there is also slight discontinuity deflection deformation between concrete face slabs. The maximum deflection deformation of concrete face slabs is approximately 0.5 times the height of the concrete face slab at the vertical direction and 1/2 times the length of the dam axis at the horizontal direction. As the dip angle of the dam foundation increases, the law of deflection deformation of concrete face slabs is basically similar, and the discontinuous deflection deformation between concrete face slabs gradually weakens. Figure 10 shows the relationship between the deflection deformation of concrete face slabs and the dip angle of the dam foundation (normal operation period). When the dip angle of the dam foundation is 0°, 5°, 10°, 15°, and 20°, the maximum deflection deformation of concrete face slabs under the co-action of gravity and water pressure is 0.163, 0.181, 0.203, 0.235, and 0.277 m. The larger the dip angle of the dam foundation, the greater the deflection deformation of concrete face slabs, which is mainly controlled by the deflection deformation of the dam body; the deflection deformation of concrete face slabs when the dip angle of the dam foundation is 20° is approximately 1.7 times that of 0°.

**Figure 11:**
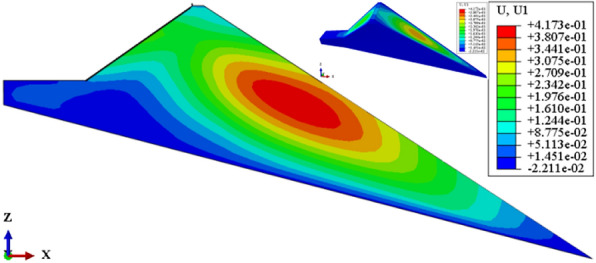
Deflection deformation contour of the dam body when the dip angle of the dam foundation is 15° (unit: m).

**Figure 12:**
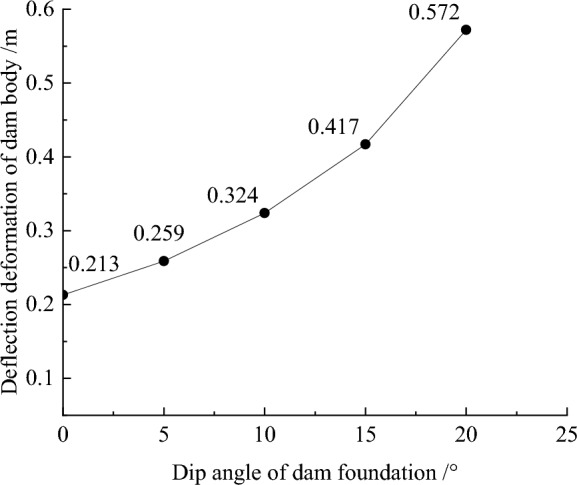
Relationship between the deflection deformation of the dam body and the dip angle of the dam foundation (unit: m).

Figure 11 shows the deflection deformation contour of the dam body for CRFD on the dam foundation with a large dip angle during the normal operation period when the dip angle of the dam foundation is 15°. The deflection deformation of the dam body is approximately elliptical. When the dam foundation is flat (the dip angle of the dam foundation is 0°), the maximum deflection deformation is approximately 1/3 times the dam height at the vertical direction and 1/4 times the length of the dam bottom downstream of the dam axis at the horizontal direction. When the dam foundation tilts, the position of maximum deflection deformation slightly decreases, and slightly shifts towards the downstream direction. As the dip angle of the dam foundation increases, the position of maximum deflection deformation is slightly lower and tends to downstream direction. Figure 12 shows the relationship between the deflection deformation of the dam body and the dip angle of the dam foundation (normal operation period). When the dip angle of the dam foundation is 0°, 5°, 10°, 15°, and 20°, the maximum deflection deformation of the dam body under the co-action of gravity and water pressure is 0.213, 0.259, 0.324, 0.417, and 0.572 m. The maximum deflection to span ratio is approximately 0.2% and less than 1.0%. The larger the dip angle of the dam foundation, the greater the deflection deformation of the dam body, the deflection deformation when the dip angle of the dam foundation is 20° is approximately three times that of 0°.

### Effect of the dip angle of the dam foundation on the structural joint of the concrete face slab

**Figure 13:**
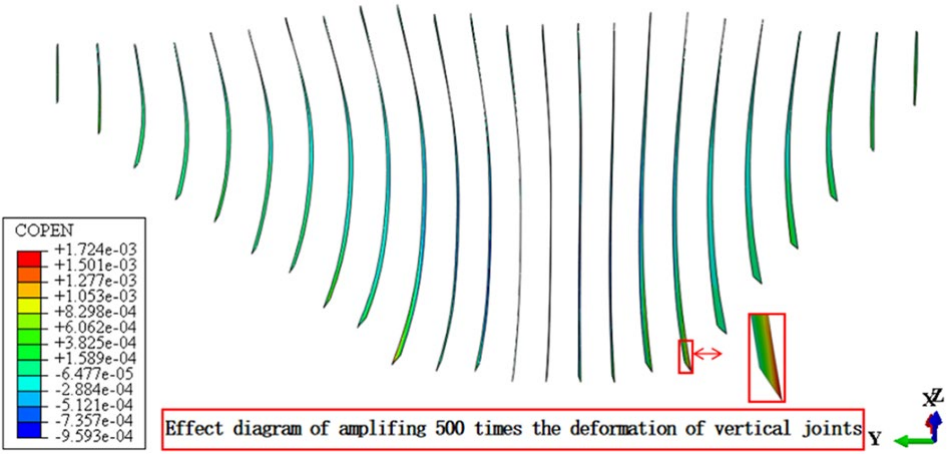
Opening deformation of vertical joints for concrete face slabs.

**Figure 14:**
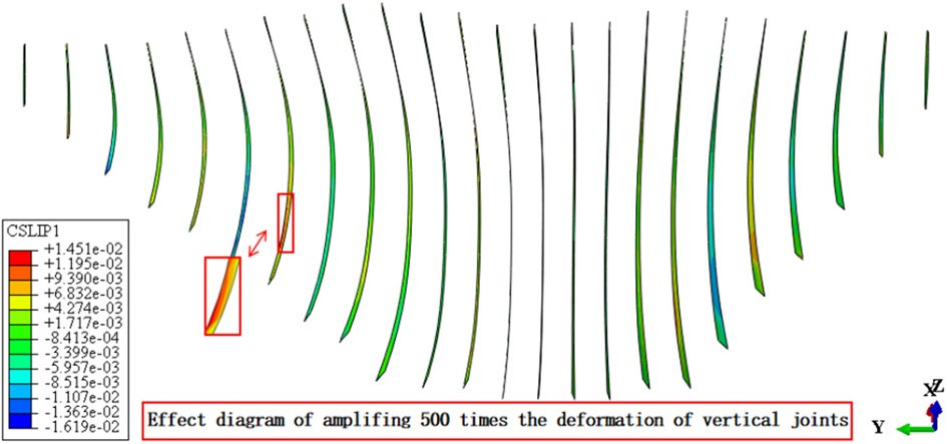
CSLIP1 deformation of vertical joints for concrete face slabs.

**Figure 15:**
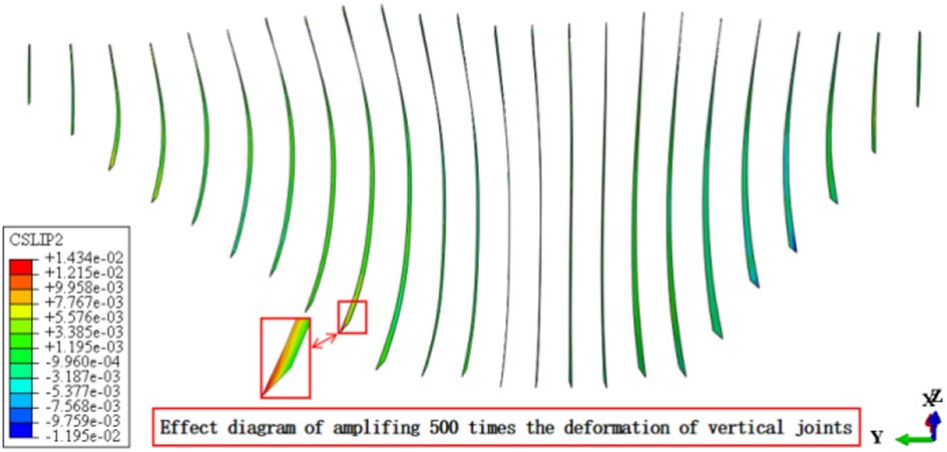
CSLIP2 deformation of vertical joints for concrete face slabs.

**Figure 16:**
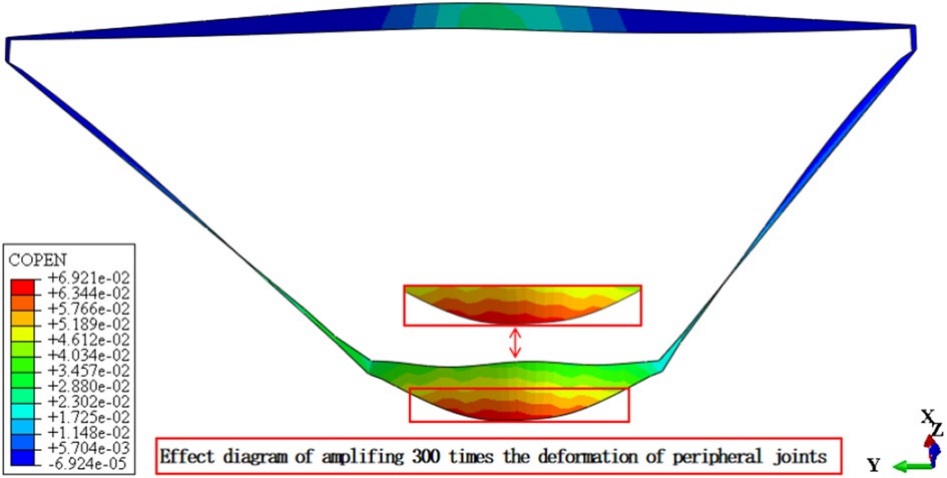
Opening deformation of peripheral joints for concrete face slabs.

**Figure 17:**
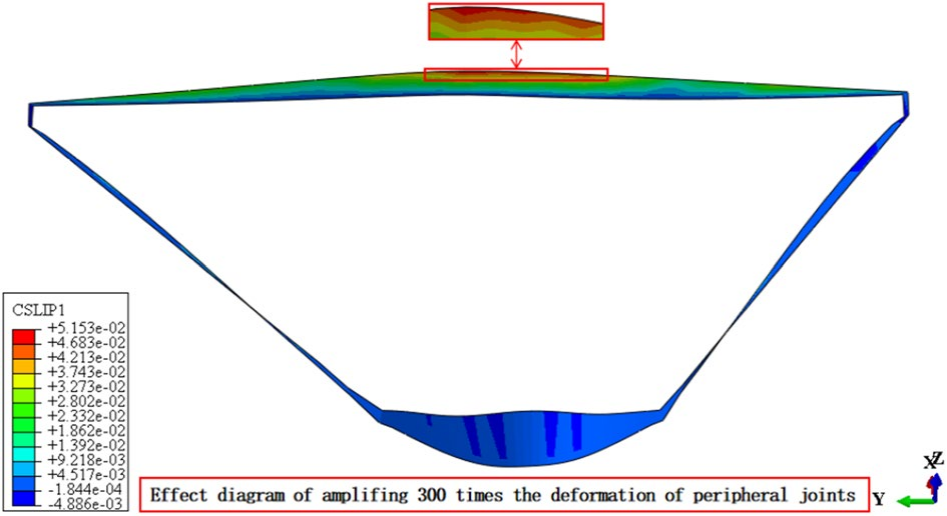
CSLIP1 deformation of peripheral joints for concrete face slabs.

**Figure 18:**
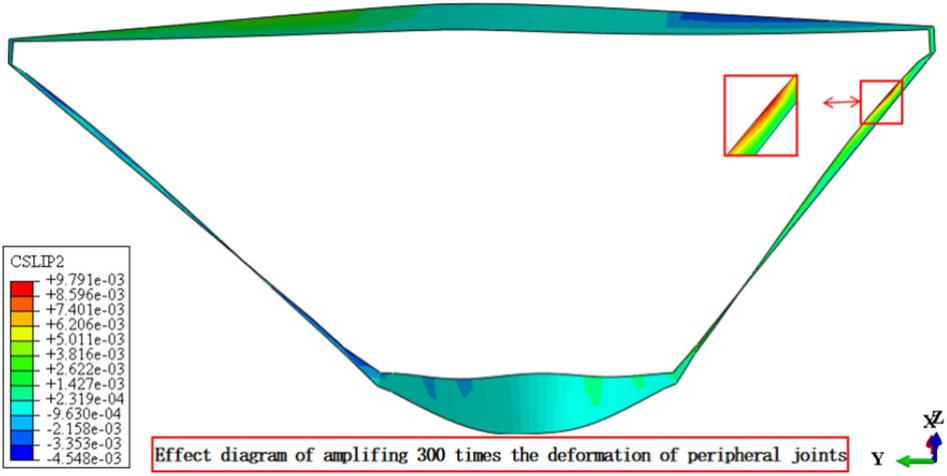
CSLIP2 deformation of peripheral joints for concrete face slabs.

Figures 13, 14, 15, 16, 17 and 18 show the three-dimensional deformation contour of structural joints of concrete face slabs for CFRD on the dam foundation with a large inclination angle during the normal operation period when the dip angle of the dam foundation is 15°. The deformation of structural joints for concrete face slabs is shown in Table 3 when the dip angle of the dam foundation is 0°, 5°, 10°, 15°, and 20°. Under the co-action of gravity and water pressure, the concrete face slab produces longitudinal deformation towards the center of the river valley, transverse deformation towards the downstream direction, and vertical deformation towards the dam foundation. At the same time, the maximum three-dimensional deformation of vertical joints for concrete face slabs is basically located near the slopes of both banks due to the constraints of the river valley. The CSLIP1 (CSLIP1 is the longitudinal relative slip deformation of the structural joints of concrete face slabs.) deformation for the vertical joints of concrete face slabs is the largest, followed by CSLIP2 (CSLIP2 is the lateral relative slip deformation of the structural joints of concrete face slabs.) deformation, and the opening deformation for the vertical joint of concrete face slabs is the smallest, all of which increase with the increase of the dip angle of the dam foundation. When the dip angle of the dam foundation is 20°, the maximum opening deformation, CSLIP2 deformation, and CSLIP1 deformation are 0.173, 1.521, and 1.524 cm, respectively. The maximum opening deformation area for the peripheral joints of concrete face slabs is located at the bottom middle part of the toe board, the maximum CSLIP2 deformation area for the peripheral joints of concrete face slabs is located at the upper middle part of the toe board for the right bank (at the steeper side of the bank slope), the maximum CSLIP1 deformation area for the peripheral joints of concrete face slabs is located at the bottom middle part of the parapet wall. The maximum opening deformation for the peripheral joints of concrete face slabs is the largest, followed by CSLIP1 deformation, and the maximum CSLIP2 deformation for the peripheral joints of concrete face slabs is the smallest, all of which increase with the increase of the dip angle of the dam foundation. When the dip angle of the dam foundation is 20°, the maximum opening deformation, CSLIP2 deformation, and CSLIP1 deformation are 7.102, 1.105, and 5.581 cm, respectively. In summary, the three-dimensional deformation for the peripheral joints of concrete face slabs is greater than that of vertical joints. It is necessary to pay special attention to the damage that may be caused by the excessive deformation of the peripheral joints of concrete face slabs, and at the same time, whether the concrete face slab produces structural damage such as compression or tension.

**Table 3:** Statistical analysis of extreme deformation values for structural joints (cm).

Dip angle of dam foundation	Type	COPEN		CSLIP2		CSLIP1	
		Maximum	Position	Maximum	Position	Maximum	Position
0°	Vertical joints	0.162	At the connection between the right bottom corner of the face slab and the toe board	0.989	At the connection between the left bottom corner of the face slab and the toe board	1.105	At the connection between the left bottom corner of the face slab and the toe board (upward)
	Peripheral joints	6.045	At the connection between the bottom middle part of the toe board and the face slab	0.60	At the connection between the middle upper part of the toe board for the right bank and the face slab	1.801	At the middle connection between the face slab and the parapet wall
5°	Vertical joints	0.164	Same as 0°	1.104	Same as 0°	1.217	Same as 0°
	Peripheral joints	6.358	Same as 0°	0.721	Same as 0°	2.031	Same as 0°
10°	Vertical joints	0.169	Same as 0°	1.250	Same as 0°	1.312	Same as 0°
	Peripheral joints	6.789	Same as 0°	0.763	Same as 0°	2.120	Same as 0°
15°	Vertical joints	0.172	Same as 0°	1.434	Same as 0°	1.451	Same as 0°
	Peripheral joints	6.921	Same as 0°	0.980	Same as 0°	5.153	Same as 0°
20°	Vertical joints	0.173	Same as 0°	1.521	Same as 0°	1.524	Same as 0°
	Peripheral joints	7.102	Same as 0°	1.105	Same as 0°	5.581	Same as 0°

### Effect of the dip angle of dam foundation on the anti-sliding stability of dam slope

**Figure 19:**
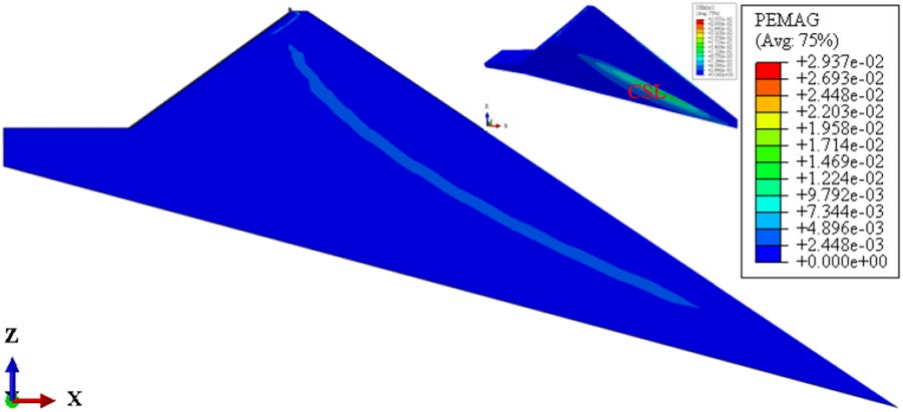
Plastic strain contour of the dam body when the dip angle of the dam foundation is 15°.

**Figure 20:**
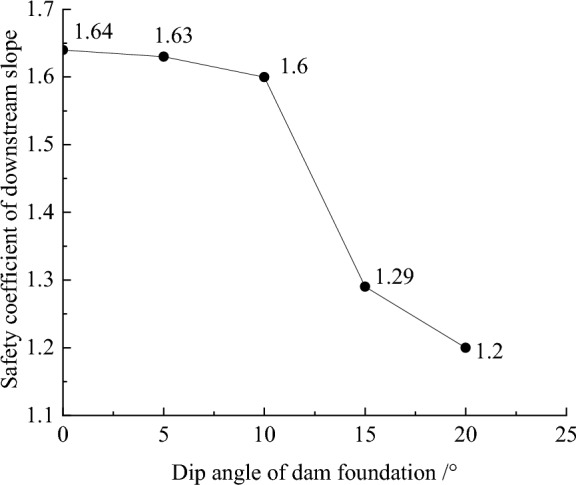
Relationship between the safety coefficient of dam slope and the dip angle of the dam foundation.

Figure 19 shows the plastic strain contour of CFRD on the dam foundation with a large dip angle during the normal operation period when the dip angle of the dam foundation is 15°. The distribution for the plastic strain of the dam body is approximately inclined with a bowl shape. When the dam foundation is flat (the dip angle of the dam foundation is 0°), the outlet position for the slip surface of the dam slope is approximately 1/2 times the dam height, and the area is small. When the dam foundation is inclined, the outlet position for the slip surface of the dam slope decreases, and the area increases. The larger the dip angle of the dam foundation, the lower the outlet position of the slip surface, the closer the bank slope, and the larger the area. Figure 20 shows the relationship between the safety coefficient of the dam slope and the dip angle of the dam foundation (normal operation period). When the dip angle of the dam foundation is 0°, 5°, 10°, 15°, and 20°, the safety coefficient of the dam slope under the co-action of gravity and water pressure is 1.64, 1.63, 1.60, 1.29, and 1.2. The larger the dip angle of the dam foundation, the smaller the safety coefficient of the dam slope due to the increase of slip deformation (settlement deformation and deflection deformation). The safety coefficient of the dam slope is less than the minimum value required by codes, i.e., 1.30 during the completion period and 1.50 during the normal storage period. The CFRDs on dam foundations with large dip angles have hidden dangers of landslide damage. Therefore, it is urgent to explore the mechanism for the initiation of slip deformation for CFRDs on dam foundations with dip angles and propose the design measures of physical mechanic and geometric structure that can reduce the slip deformation of concrete face slabs and dam body to improve the anti-sliding stability of the dam slope.

The slope along the river of the dam foundation for the CFRDs of normal hydropower stations is relatively gentle. The maximum settlement deformation of the dam body under the action of gravity is approximately located near the dam axis. The deflection deformation of the dam body under the co-action of gravity and water pressure is relatively small. However, the CFRDs in the upper reservoirs of PSPSs are usually located on the mountaintop or mountainside of alpine and canyon regions. The slope along river of dam foundation is steep (large dip angle). The maximum settlement deformation of the dam body under the action of gravity tends to be downstream. The deflection deformation of the dam body under the co-action of gravity and water pressure is relatively large. Therefore, the CFRDs for the upper reservoirs of PSPSs are prone to produce slip deformation (characterized by settlement deformation and deflection deformation), and the anti-sliding stability of the dam slope is poor.

## The mechanism for the slip deformation of CFRDs on dam foundations with large dip angles

**Figure 21:**
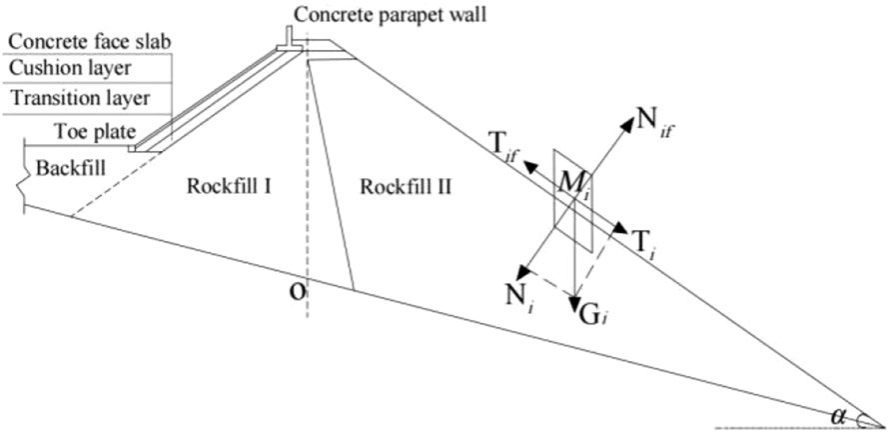
Schematic diagram for the partial force balance of the downstream dam slope.

The CFRDs on dam foundations with large dip angles inevitably produce settlement deformation and deflection deformation (slip deformation) under the co-action of gravity and water pressure. Moreover, the larger the dip angle of the dam foundation, the greater the slip deformation of concrete face slabs and dam body, and the poorer the anti-sliding stability of the dam slope, which is more unfavorable for the operation safety of CFRDs for the upper reservoirs of PSPSs. In view of this, the schematic diagram for the partial force balance of the downstream dam slope for CFRD on the dam foundation with a dip angle is established, as shown in Fig. 21. The mechanism for the slip deformation of CFRDs on dam foundations with dip angles is explored to provide theoretical basis for proposing improvement measures to reduce the slip deformation of concrete face slabs and dam body to improve the anti-sliding stability of the dam slope. Any rockfill unit *M*_*i*_ that its side surface is vertical and its bottom surface is parallel to the dam slope is taken from the free surface of the downstream dam slope. If the influence of forces on both sides and the cohesion of rockfill (which is relatively small) are ignored, then the self-weight of rockfill unit *M*_*i*_ is *G*_*i*_, and the internal friction angle of rockfill unit *M*_*i*_ is *φ*. The components of the gravity of rockfill unit *M*_*i*_ at the tangential and normal directions of the downstream dam slope are shown in Eq. (3).

3 $$ \left\{ {\begin{array}{*{20}c} {G_{i} {\text{sin}}\alpha = T_{i} } \\ {G_{i} {\text{cos}}\alpha = N_{i} } \\ \end{array} } \right. $$

where *G*_*i*_ is the gravity of rockfill unit *M*_*i*_;* α* is the dip angle of the dam foundation; *T*_*i*_ is the tangential component of the gravity of rockfill unit *M*_*i*_ (sliding force); and *N*_*i*_ is the component of the gravity of rockfill unit *M*_*i*_. The anti-sliding force *T*_*if*_ is provided to prevent the sliding of rockfill unit *M*_*i*_, as shown in Eq. (4).

4 $$ T_{if} = N_{i} \tan \varphi = G_{i} {\text{cos}}\alpha \tan \varphi $$

Therefore, the safety coefficient *K* of rockfill unit *M*_*i*_ is shown in Eq. (5).

5 $$ K = \frac{{T_{if} }}{{T_{i} }} = \frac{{G_{i} {\text{cos}}\alpha \tan \varphi }}{{G_{i} {\text{sin}}\alpha }} = \frac{\tan \varphi }{{\tan \alpha }} $$

Equation (4) shows that the tangential component of the gravity of rockfill unit *M*_*i*_ along the slope surface, the sliding force of the dam body, and the slip deformation of the dam body increases with the increase of the dip angle *α* for the dam foundation. In addition, the gravity component of rockfill unit *M*_*i*_ decreases along the normal direction of the slope surface, which reduces the driving force for providing the anti-sliding force of rockfill unit *M*_*i*_. It is equivalent to weakening the constraint effect of the dam body. The slip deformation of concrete face slabs and dam body will increase further. When the dip angle of the dam foundation increases to the same angle as the internal friction angle of the rockfill, the safety coefficient of the dam slope is equal to 1.0, and the dam slope is at the state of ultimate equilibrium (the sliding force and anti-sliding force are equal.). When the dip angle of the dam foundation continues to increase, the dam slope will produce sliding deformation (landslip). In summary, the large sliding force and small anti-sliding force are the fundamental reasons that CFRDs on dam foundations with large dip angles are prone to produce slip deformation (poor anti-sliding stability of the dam slope).

## Measures proposed for improving the anti-sliding stability of CFRDs on dam foundations with large inclinations

Aiming at the problem that CFRDs on dam foundations with large dip angles are prone to produce slip deformation due to large sliding forces and small anti-sliding forces, the design measure for the addition of the pressure slope is proposed to reduce the slip deformation of concrete face slabs and dam body and improve the anti-sliding stability of the dam slope. This method is feasible in practical engineering, such as Fukang PSPS in China. The pressure slope not only improves the anti-sliding force of the dam slope but also fully utilizes the excavation materials of the upper reservoir basin to achieve the better balance between excavation and filling. Then, the influences of the height or width of the pressure slope platform and the cohesion or internal friction angle of the pressure slope on the slip deformation of concrete face slabs and dam body and the safety factor of the dam slope are studied. Finally, the parameters for the physical mechanic and geometric structure of the pressure slope that are suitable for the construction of CFRDs for the upper reservoirs of PSPSs are recommended. It should be noted that the addition of the pressure slope can reduce the deformation of the peripheral and vertical seams of concrete face slabs, but the impact is not significant yet, so it will not be further elaborated in the following text.

### Geometric structural measures to improve the anti-sliding stability of the dam slope

**Figure 22:**
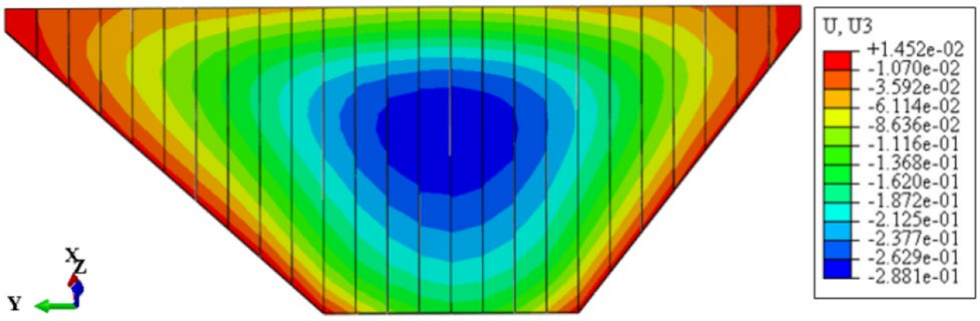
Settlement deformation contour of concrete face slabs with pressure slope when the dip angle of the dam foundation is 15° (unit: m).

**Figure 23:**
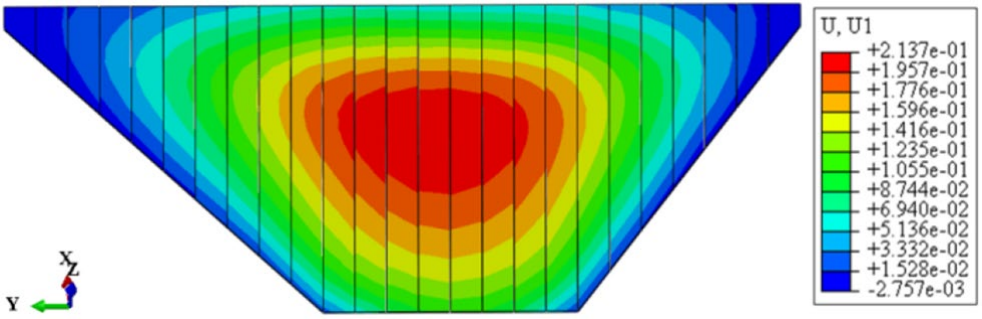
Deflection deformation contour of concrete face slabs with pressure slope when the dip angle of the dam foundation is 15° (unit: m).

**Figure 24:**
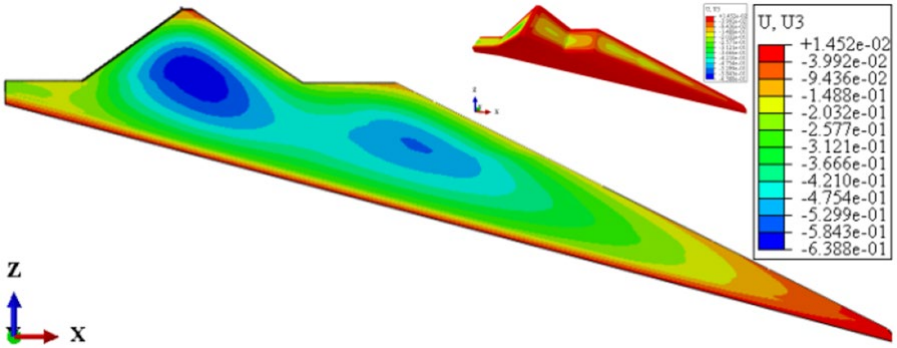
Settlement deformation contour of the dam body with pressure slope when the dip angle of the dam foundation is 15° (unit: m).

**Figure 25:**
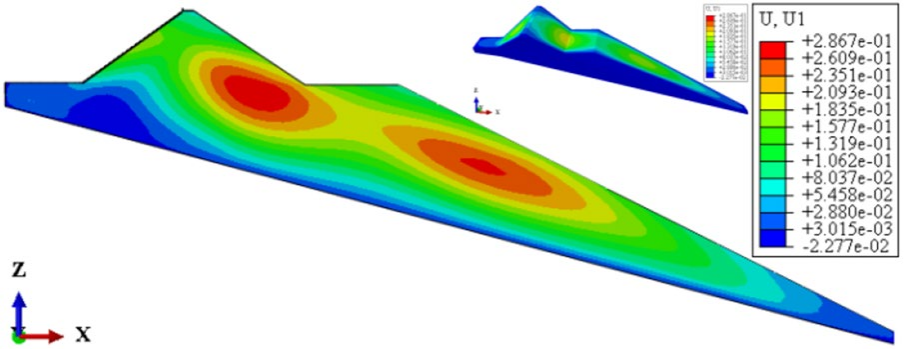
Deflection deformation contour of the dam body with pressure slope when the dip angle of the dam foundation is 15° (unit: m).

**Figure 26:**
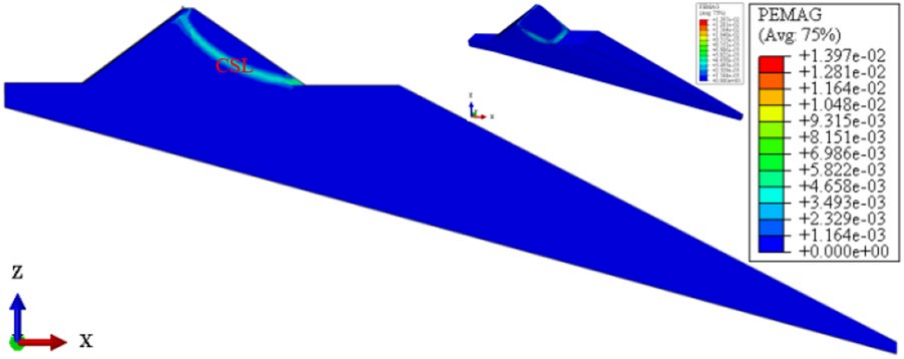
Plastic strain contour of the dam body with pressure slope when the dip angle of the dam foundation is 15°.

**Figure 27:**
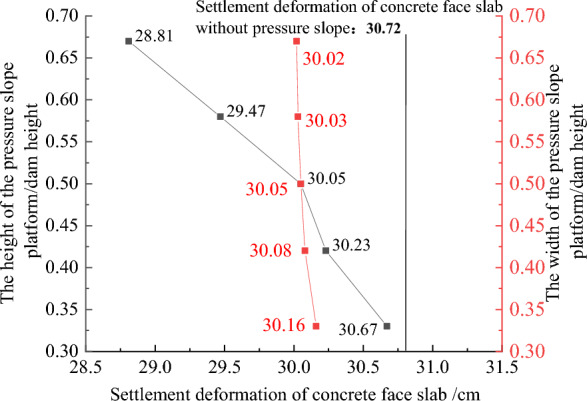
Relationships between the settlement deformation of concrete face slab swith pressure slope and the height and width of the pressure slope platform (unit: cm).

**Figure 28:**
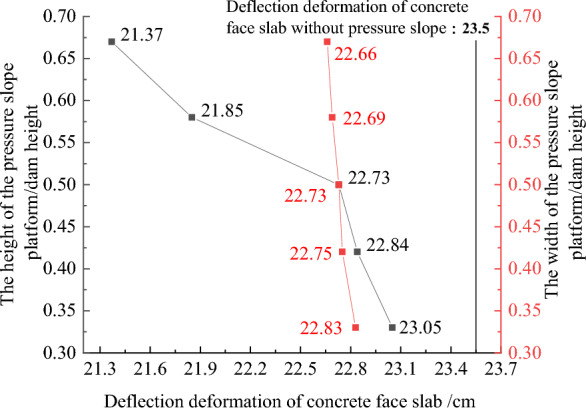
Relationships between the deflection deformation of concrete face slabs with pressure slope and the height and width of the pressure slope platform (unit: cm).

**Figure 29:**
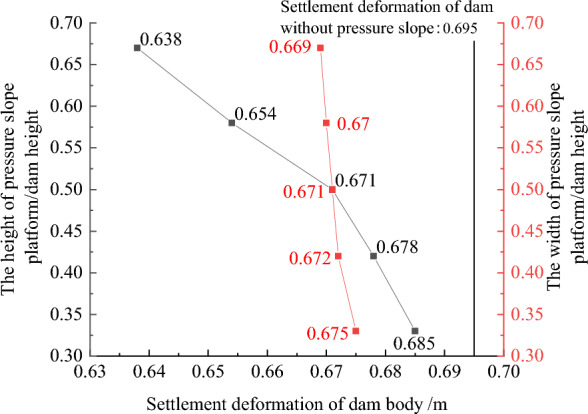
Relationships between the settlement deformation of the dam body with pressure slope and the height and width of the pressure slope platform (unit: m).

**Figure 30:**
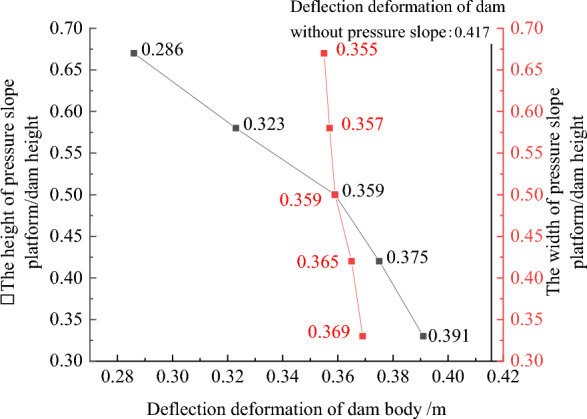
Relationships between the deflection deformation of the dam body with pressure slope and the height and width of the pressure slope platform (unit: m).

**Figure 31:**
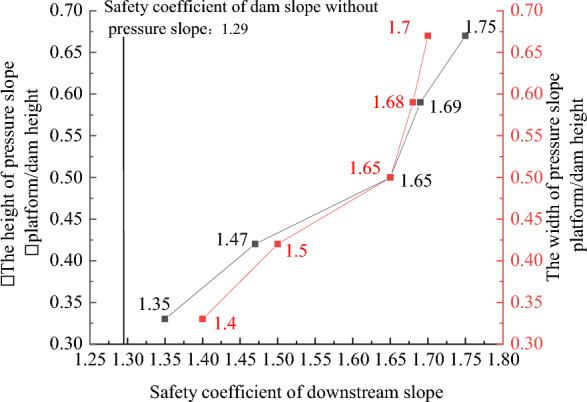
Relationships between the safety coefficient of the downstream dam slope with pressure slope and the height and width of the pressure slope platform (unit: m).

Figures 22, 23, 24, 25, and 26 show the settlement deformation, deflection deformation, and plastic strain contour of concrete face slabs and dam body with pressure slope for CFRD on the dam foundation with a large dip angle during the normal operation period when the dip angle of the dam foundation is 15°. The distributions of settlement deformation, deflection deformation, and plastic strain of concrete face slabs and dam body for the main dam are similar to those of the non-pressure slope. The difference is that the maximum settlement deformation and deflection deformation of the dam body with pressure slope are shifted upstream (closer to the dam axis) compared with those of non-pressure slope, and there are two extreme points of the settlement deformation and deflection deformation of the dam body with pressure slope (one for main dam body, and one for pressure slope). Figures 27, 28, 29, 30, and 31 show the relationships between the maximum settlement deformation, deflection deformation of concrete face slabs and dam body, the minimum safety factor of the downstream dam slope and the height and width of the pressure slope platform. When the height and width of the pressure slope platform are 1/3, 2/5, 1/2, 4/7, and 2/3 times the maximum height of the main dam, the maximum settlement deformation of concrete face slabs is 30.67, 30.23, 30.05, 29.47, 28.81 cm and 30.16, 30.08, 30.05, 30.03, 30.02 cm, respectively, the maximum deflection deformation of concrete face slabs is 23.05, 22.84, 22.73, 21.85, 21.37 cm, and 22.83, 22.75, 22.73, 22.69, 22.66 cm, respectively; the maximum settlement deformation of the dam body is 0.685, 0.678, 0.671, 0.654, 0.638 m and 0.675, 0.672, 0.671, 0.670, 0.669 m, respectively, the maximum deflection deformation of the dam body is 0.391, 0.375, 0.359, 0.323, 0.286 m and 0.369, 0.365, 0.359, 0.357, 0.355 m, respectively; the minimum safety factor of the downstream dam slope is 1.35, 1.47, 1.65, 1.69, 1.75, and 1.40, 1.50, 1.65, 1.68, 1.70, respectively. When the height and width of the pressure slope platform exceed 1/2 of the maximum height of the main dam, the minimum safety factor of the downstream dam slope exceeds the minimum value of 1.50 required by codes. The influences of the height of the pressure slope platform on the settlement deformation and deflection deformation of concrete face slabs and dam body and the minimum safety factor of the downstream dam slope are significantly greater than those of the width of the pressure slope platform. Moreover, the settlement deformation and deflection deformation of concrete face slabs and dam body with pressure slope are significantly reduced, and the safety factor of the downstream dam slope is significantly increased by comparing it with non-pressure slope. The higher and wider the pressure slope platform, the smaller the settlement deformation and deflection deformation of concrete face slabs and main dam body, the greater the safety factor of the downstream dam slope, and the better the anti-sliding stability of the dam slope. It is expected that the larger the dip angle of the dam foundation, the longer the length of the dam axis, and the greater the effect for the increase of the height and width of the pressure slope platform on reducing the slip deformation of concrete face slabs and dam body, and improving the anti-sliding stability of the downstream dam slope.

In summary, the addition of the pressure slope can not only significantly reduce the slip deformation (settlement deformation and deflection deformation) of concrete face slabs and dam body and significantly improve the anti-sliding stability of the downstream dam slope, but also reduce environmental pollution and achieve the balance between excavation and filling. It should be noted that the greater the height and width of the pressure slope platform, the better the decrease effect for the slip deformation of concrete face slabs and dam body. However, when the height and width of the pressure slope platform reach a certain value, if the height and width of the pressure slope platform are further increased, the effect that reduces slip deformation and improves anti-sliding stability will no longer be significant. Considering the impact of construction costs and operational safety, it is recommended to take the height and width of the pressure slope platform as approximately 1/2 times the maximum height of the main dam.

### Physical mechanical measures to improve the anti-sliding stability of the dam slope

**Figure 32:**
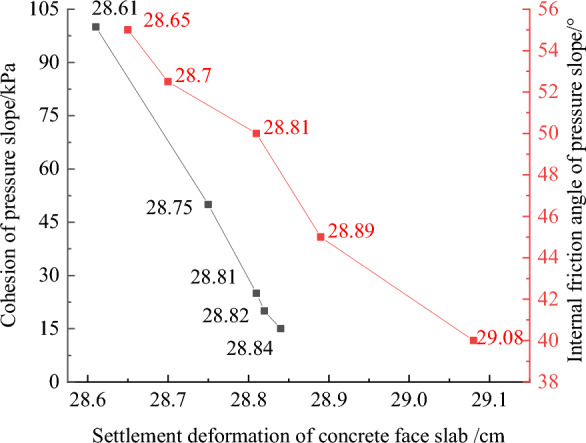
Relationships between the settlement deformation of concrete face slabs with pressure slope and the cohesion and internal friction angle of the pressure slope (unit: cm).

**Figure 33:**
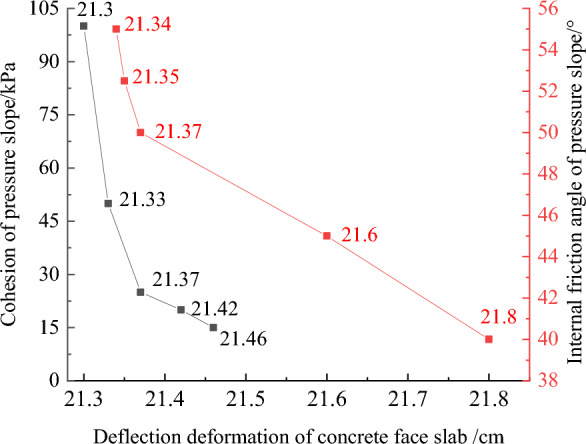
Relationships between the deflection deformation of concrete face slabs with pressure slope and the cohesion and internal friction angle of the pressure slope (unit: cm).

**Figure 34:**
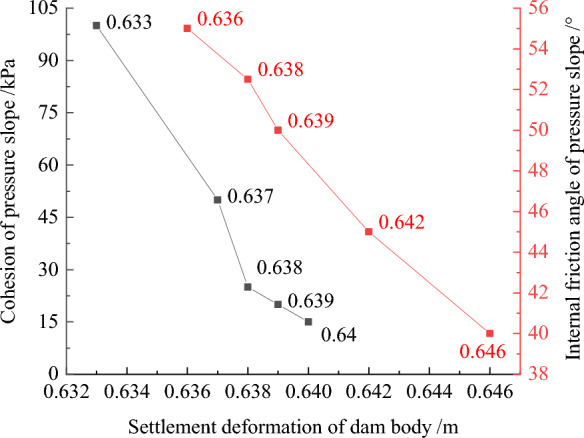
Relationships between the settlement deformation of the dam body with pressure slope and the cohesion and internal friction angle of the pressure slope (unit: m).

**Figure 35:**
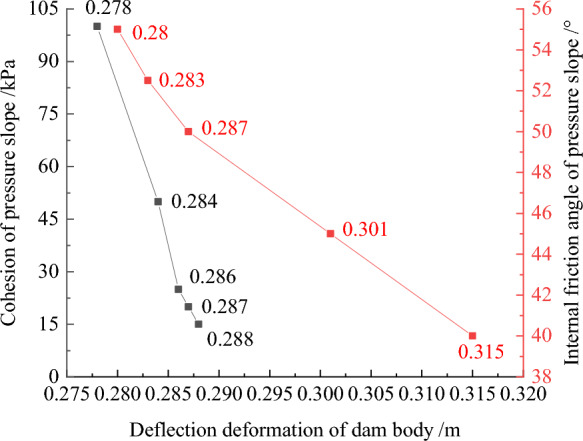
Relationships between the deflection deformation of the dam body with pressure slope and the cohesion and internal friction angle of the pressure slope (unit: m).

**Figure 36:**
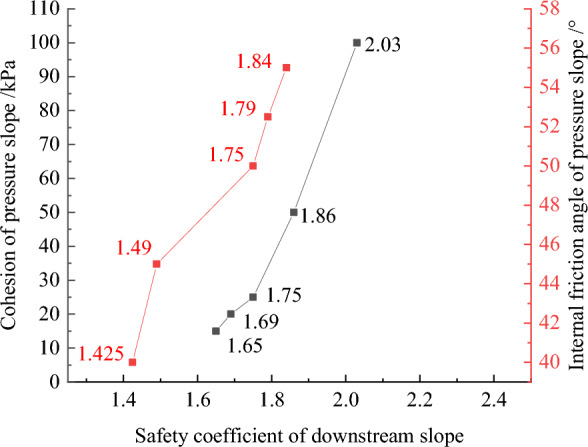
Relationships between the safety coefficient of the downstream dam slope with pressure slope and the cohesion and internal friction angle of the pressure slope.

Figures 32, 33, 34, 35, and 36 show the relationships between the maximum settlement deformation, deflection deformation of concrete face slabs and dam body with pressure slope, the minimum safety factor of the downstream dam slope and the cohesion and internal friction angle of the pressure slope, respectively. The height and width of the pressure slope platform are taken as 2/3 and 1/2 times the maximum height of the main dam, respectively. When the cohesion and internal friction angle of the pressure slope are 15, 20, 25, 50, 100 kPa and 40°, 45°, 50°, 52.5°, 55°, the maximum settlement deformation of concrete face slabs is 28.84, 28.82, 28.81, 28.75, 28.61 cm and 29.08, 28.89, 28.81, 28.70, 28.65 cm; the maximum deflection deformation of concrete face slabs is 21.46, 21.42, 21.37, 21.33, 21.30 cm and 21.80, 21.60, 21.37, 21.35, 21.34 cm; the maximum settlement deformation of the dam body is 0.40, 0.639, 0.638, 0.637, 0.633 m and 0.646, 0.642, 0.639, 0.638, 0.636 m; the maximum deflection deformation of the dam body is 0.288, 0.287, 0.286, 0.284, 0.278 m and 0.315, 0.301, 0.287, 0.283, 0.280 m; and the minimum safety factor (coefficient) of the downstream dam slope is 1.65, 1.69, 1.75, 1.86, 2.03, and 1.425, 1.49, 1.75, 1.79, 1.84, respectively, which exceeds the minimum value of 1.50 required by codes. The influence of the cohesion of the pressure slope on the settlement deformation and deflection deformation of concrete face slabs and dam body, as well as the safety factor of the downstream dam slope, is less than that of the internal friction angle of the pressure slope. The settlement and deflection deformation of concrete face slabs and dam body are both reduced, and the safety coefficient of the downstream dam slope is significantly increased by comparing with those of non-pressure slope. Moreover, the greater the cohesion and internal friction angle of the pressure slope, the smaller the settlement and deflection deformation of concrete face slabs and dam body, the greater the safety coefficient of the downstream dam slope, and the better the anti-sliding stability of the dam slope. It is expected that the larger the dip angle of the dam foundation, the longer the dam axis, and the greater the effect for the increase of the cohesion and internal friction angle of the pressure slope on reducing the slip deformation of concrete face slabs and dam body and improving the anti-sliding stability of the dam slope.

In summary, the increase of the height and width of the pressure slope platform can reduce the slip deformation of concrete face slabs and dam body and improve the anti-sliding stability of the dam slope, the increase of the cohesion and internal friction angle of the pressure slope can achieve expected effects as well. The greater the cohesion and internal friction angle of the pressure slope, the better the effect that reduces the slip deformation of concrete face slabs and dam body. However, when the cohesion and internal friction angle of the pressure slope reach a certain value, if the cohesion and internal friction angle of the pressure slope are further increased, the effect that reduces slip deformation and improves anti-sliding stability will no longer be significant. Considering factors such as construction cost and operational safety, it is recommended that the density (cohesion and internal friction angle) of the pressure slope be equivalent to that of the main dam’s rockfill material.

## Conclusions

Aiming at the problem that CFRDs on dam foundations with large dip angles are prone to produce slip deformation, the influence of the dip angle of the dam foundation on slip deformation is studied. The mechanism for the initiation of the slip deformation of CFRDs on dam foundations with large angles is revealed. The design measures of physical mechanic and geometric structure are proposed to reduce slip deformation. The main conclusions are as follows:

- The slip deformation of CFRDs on dam foundations with large angles is characterized by settlement deformation and deflection deformation. The settlement deformation and deflection deformation of concrete face slabs and dam body, as well as the three-dimensional deformation for the structural joints of concrete face slabs, are increased with the dip angle of the dam foundation. The safety factor of the downstream dam slope decreases with the increase of the dip angle of the dam foundation. When the dip angle of the dam foundation exceeds 15°, the safety factor of the dam slope is 1.29, which is less than the minimum value of 1.50 required by codes. The CFRDs on dam foundations with large dip angles have the risk of instability.
- The larger the dip angle of the dam foundation, the greater the component of the gravity of rockfill along the slope direction (the greater the sliding force), and the smaller the friction force between dam foundation and rockfill (the smaller the anti-sliding force and the weaker the boundary constraint ability). Therefore, the larger sliding forces and smaller anti-sliding forces are the fundamental reasons that CFRDs on dam foundations with large dip angles are prone to produce slip deformation.
- The addition of the pressure slope can significantly reduce the slip deformation of concrete face slabs and dam body, but it has little effect on the three-dimensional deformation of the structural joints of concrete face slabs. Moreover, the addition of the pressure slope can significantly improve the anti-sliding stability of the downstream dam slope. The greater the height and width of the pressure slope platform, the greater the cohesion and internal friction angle, the smaller the settlement and deflection deformation of concrete face slabs and dam body, and the greater the safety coefficient of the dam slope (the stronger the ability of the dam slope to resist sliding). It is recommended that the height and width of the pressure slope platform be taken as 1/2 times the maximum height of the main dam and the compaction degree (cohesion and internal friction angle) of the pressure slope be basically equivalent to the degree of compaction of the rockfill of the main dam.

## Data Availability

All associated data have been presented in the manuscript which are available from the corresponding author on reasonable request.
